# MicroRNA-93-5p may participate in the formation of morphine tolerance in bone cancer pain mouse model by targeting Smad5

**DOI:** 10.18632/oncotarget.10524

**Published:** 2016-07-11

**Authors:** Wen-Feng Xiao, Yu-Sheng Li, Wei Lou, Ting Cai, Shun Zhang, Xiao-Ying Hu, Xing-Wang Zhang, Wei Luo

**Affiliations:** ^1^ Department of Orthopaedics, Xiangya Hospital, Central South University, Changsha 410008, China; ^2^ Department of Pain Treatment, Ningbo No.2 Hospital, Ningbo 315010, China; ^3^ Department of Emergency Internal Medicine, Ningbo No.2 Hospital, Ningbo 315010, China; ^4^ Stem Cell Laboratory, Ningbo No.2 Hospital, Ningbo 315010, China; ^5^ Department of Nephropathy Dialysis Center, Shuguang Hospital Affiliated to Shanghai University of Traditional Chinese Medicine, Shanghai 201203, China; ^6^ Division of Pharmaceutics, College of Pharmacy, Jinan Unversity, Guangzhou 510632, P.R. China

**Keywords:** microRNA-93-5p, Smad5, bone cancer pain, morphine tolerance, paw mechanical withdrawal threshold

## Abstract

**Objective:**

In this study, we aim to find out the role of microRNA-93-5p (miR-93) and Smad5 in morphine tolerance in mouse models of bone cancer pain (BCP).

**Results:**

At 7 days after injection of morphine, the PMWT showed no significant difference between the morphine model group and the saline model group (*P* < 0.05), suggesting that morphine tolerance had formed in the morphine model group. The morphine model group had higher miR-93 expression and lower Smad5 mRNA expression than the saline model group. *Smad5* is a downstream target gene of miR-93. At 7, 9 and 14 days after injection of lentiviruses, the L/anti-miR-93 group had the lowest PMWTs, while the Smad5 shRNA group presented the highest PMWTs among these five groups (all *P* < 0.05).

**Methods:**

We built mouse models of BCP and morphine tolerance and recorded 50% PMWT. After 6 days of modeling, we set saline control group, morphine control, saline model group and morphine model group (morphine tolerance emerged). We performed luciferase reporter gene assay to verify the relation between miR-93 and Smad5. After lentivirus transfection, the mice with morphine tolerance were assigned into L/anti-miR-93 group, Smad5 shRNA group, L/anti-miR-93 + Smad5 shRNA group, blank group and PBS control group. RT-qPCR, Western Blot assay and immumohistochemical staining were performed to observe the changes of miR-93 and *Smad5*.

**Conclusion:**

Up-regulation of miR-93 may contribute to the progression of morphine tolerance by targeting Smad5 in mouse model of BCP.

## INTRODUCTION

Bone is the most frequently site to be affected through cancer metastasis which has been implicated in lung cancer, breast cancer, as well as hepatocellular carcinoma due to the prevalence of these diseases. At postmortem examination, up to 70% of patients dying of these cancers presents bone metastatic [1–3]. Also, in the United States, it has been estimated that tumor metastasis to the bone affects more than 400,000 persons each year [2]. This bone metastasis included a cycle of tumor growth, bone destruction, and formation of woven bone begins, inevitably bringing about considerable pain, that is, bone cancer pain (BCP) [4]. Bone cancer pain is prevalent among cancer patients and can have a devastating effect on their quality of life [5]. Morphine is a conventionally-selected pure μ-agonist opioid for pain relief in cancer patients [6]. Unfortunately, prolonged take-in of morphine may lead to cellar or molecular adaptions and therewith produce morphine tolerance [7, 8]. In this context, for more satisfactory pain relief in cancer patients, more efforts should be made to figure out the mechanism of morphine tolerance at the molecular level.

MicroRNA-93-5p (miR-93) has also been suggested as a contributing factors in disease progression trough regulation of it downstream targets, including P21, cyclin B1, ERBB2, Akt3, SOX4, PTEN, STAT3, vascular endothelial growth factor A and Smad7 [9–13]. In lung cancer, miR-93 may target LATS2 to enhance metastasis or downregulate NEDD4L to promote TGF-β-induced epithelial-to-mesenchymal transition, an important mechanism for cancer metastasis [14, 15]. Using certain biology software, we find that Smad5 is also a target gene of miR-93. As suggested, bone morphogenetic protein (BMP)/Smad signaling pathway promote the vascular development [16]. As a key mediator of BMP/Smad pathway, Smad5 can act as a target of microRNA to suppress the endothelial development and thereby the angiogenesis, a must for tumor progression and tumor metastases [17, 18]. Interestingly, Smad5 is a well-accepted suppressive gene in osteoclast differentiation and thereby decreases resorption pits [19]. To our knowledge, through these resorption pits, osteoclasts resorb bone, stimulating nerve fiber-rich TRPV1 or ASIC3 channels and consequently result in BCP [4]. On basis of all the facts, we speculate that miR-93 and Smad5 may correlate with the formation and progression of morphine tolerance.

In the present study, we build a BCP model with Lewis lung cancer cells, aiming to investigate the association between miR-93 and Smad5 and the effect of miR-93 and Smad5 on morphine tolerance.

## RESULTS

### PMWT in the BCP model and the morphine tolerance model

Table 1 showed the PMWTs before modeling and at 3^rd^, 6^th^ and 9^th^ day after modeling and at 1^st^, 3^rd^, 5^th^ and 7^th^ day after injection of morphine. As presented, at 6^th^ and 9^th^ day after modeling, the PMWT was significantly higher in the saline model group and in the morphine model group than that in the saline control group and in the morphine control group, correspondingly (the saline model vs. the saline control, *P* < 0.05; the morphine model vs. the morphine control, *P* < 0.05); the PMWTs in both saline model and morphine model groups were significantly lower at 6 days after treatment in comparison with pre-treatment (both *P* < 0.05). The above results suggested that BCP model was successfully built. At 7^th^ day after injection of morphine, the PMWT showed no significant difference between the morphine model group and the saline model group (*P* < 0.05), suggesting that morphine tolerance had formed in the morphine model group.

**Table 1:** Recordings of the PMWT of all groups during the establishment of BCP model and morphine tolerance model

	Pre-surgery (g)	Post-surgery (g)			After morphine injection (g)			
		3d	6d	9d	1d	3d	5d	7d
Saline control group	6.35 ± 0.57	6.42 ± 0.69	6.41 ± 0.74	6.56 ± 0.63	6.51 ± 0.77	6.62 ± 0.79	6.61 ± 0.84	6.57 ± 0.73
Morphine control group	6.38 ± 0.78	6.44 ± 0.74	6.39 ± 0.60	6.45 ± 0.73	8.48 ± 0.97*	8.64 ± 0.83*	8.03 ± 0.74*	6.65 ± 0.66
Saline model group	6.32 ± 0.65	6.08 ± 0.53	5.53 ± 0.56*^&^	3.65 ± 0.32*^&^	3.51 ± 0.37	3.67 ± 0.36	3.83 ± 0.39	4.32 ± 0.43
Morphine model group	6.30 ± 0.20	6.01 ± 0.58	5.34 ± 0.49*^&^	3.60 ± 0.35Δ^&^	7.58 ± 0.75^#^	7.98 ± 0.84^#^	5.88 ± 0.61^#^	4.40 ± 0.46

PMWT, paw mechanical withdrawal threshold; BCP, bone cancer pain

* *P* < 0.05 in comparison with the saline control group

Δ *P* < 0.05 in comparison with the morphine control group

# *P* < 0.05 in comparison with the saline model group

& *P* < 0.05 in comparison with pre-treatment.

### miR-93-5p expression and Smad5 mRNA and protein expression after morphine tolerance

**Figure 1:**
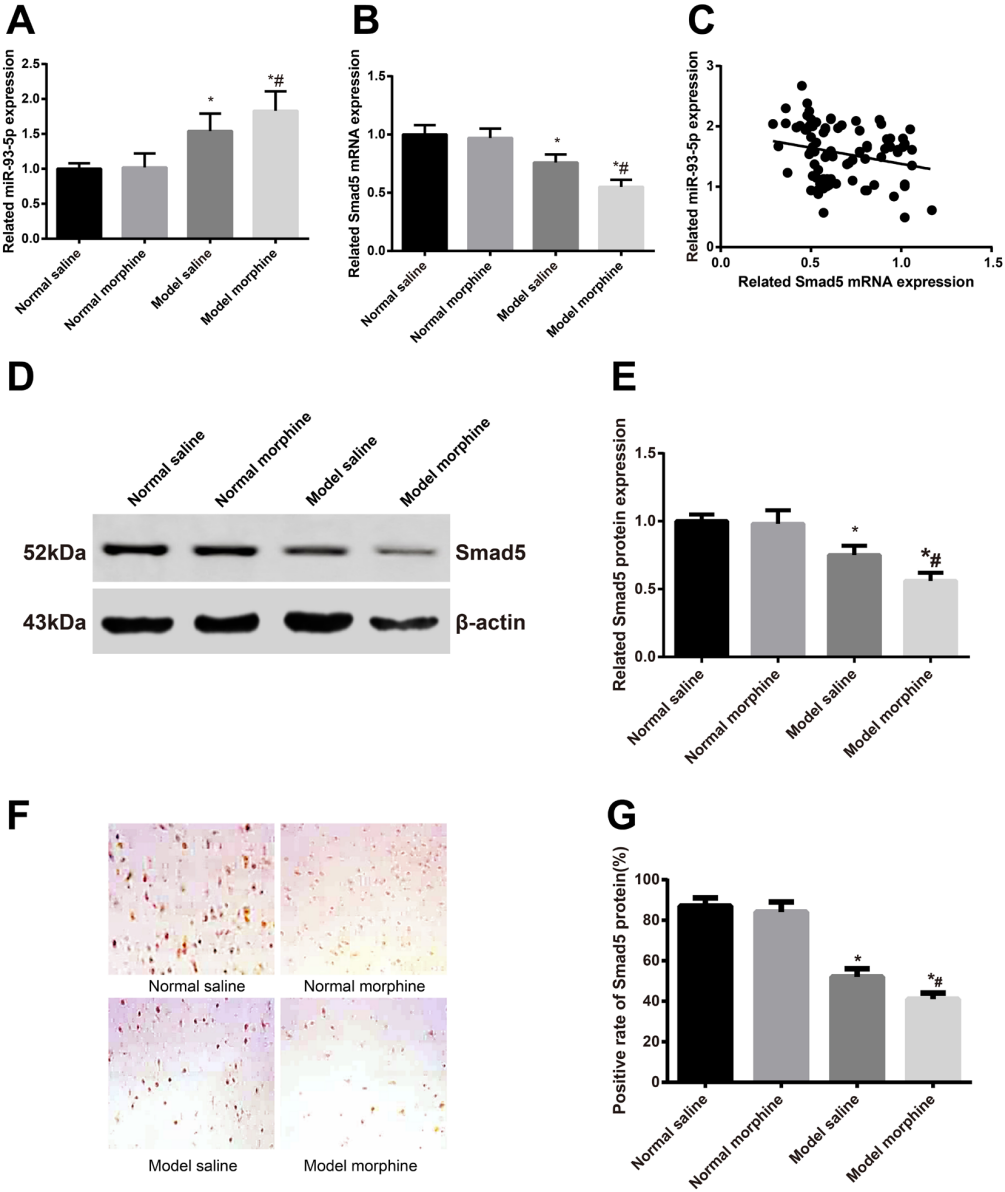
After the morphine tolerance model was built, miR-93-5p expression and Smad5 mRNA and protein expressions were detected **A.** miR-93-5p expression; **B.** Smad5 mRNA expression; **C.** miR-93-5p expression was in negative correlation with Smad5 mRNA expression; **D.** electrophoretogram of Smad5 protein by Western blot; **E.** alternations of Smad5 protein expression; **F.** Smad5 protein expression in L5 spinal marrows by immunohistochemistry; **G.** semi-quantitative Smad5 protein expression in L5 spinal marrows by immunohistochemistry. Note: *, *P* < 0.05 in comparison with the saline control group; #, *P* < 0.05 in comparison with the saline model group.

Our RT-qPCR demonstrated that, the saline model group had a higher miR-93-5p expression and lower Smad5 mRNA expression than the saline control group, but had a lower miR-93-5p expression and a higher Smad5 mRNA expression than the morphine model group (all *P* < 0.05) (Figure 1A, 1B). The above results indicated that the miR-93-5p expression was in negative association with the Smad5 mRNA expression (*P* < 0.05) (Figure 1C). Besides, we detected the Smad5 expression in the four group by Western Blot Assay (Figure 1D) and immunohistochemical staining (Figure 1E), finding that, among the four group, the saline model group had a lower Smad5 expression than the saline control group but a higher Smad5 expression than the morphine model group(both *P* < 0.05).

### miR-93 directly targets Smad5

**Figure 2:**
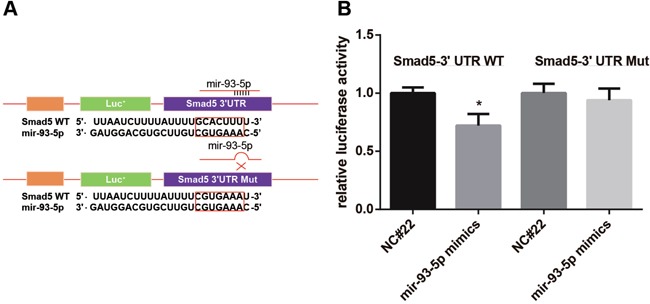
Dual luciferase reporter gene assay showed that Smad5 was a target of miR-93-5p **A.** we synthesized a target sequence WT (GCACTTT) and then a mutant sequence Mut (CGTGAAA) through site-directed mutagenesis in WT; **B.** the luciferase activity was recorded and compared.

We synthesized a sequence (GCACTTT) at target site (WT) and then a mutant sequence Mut (CGTGAAA) through site-directed mutagenesis in WT (Figure 2A) to conduct a dual luciferase reporter gene assay. The result of the luciferase reporter gene assay showed that the fluorescence intensity showed no significant difference between Group C and Group D while exhibited obvious difference between Group A and Group B (*P* < 0.05) (Figure 2B). This result suggested thatSmad5 was a target gene of miR-93-5p.

### PMWT in the mice with morphine tolerance after transfection with lentiviruses

**Figure 3:**
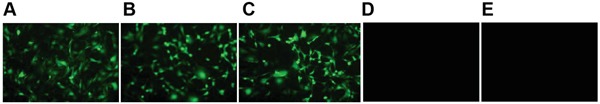
At 14 days after lentivirus transfection, L5 spinal marrows from all five groups were extracted and the transfection efficiency was evaluated **A.** represents the L/anti-miR-93-5p group; **B.** represents the Smad5 shRNA group; **C.** represents the L/anti-miR-93-5p + Smad5 shRNA group; **D.** represents the blank virus group; **E.** represents the blank control group.

**Figure 4:**
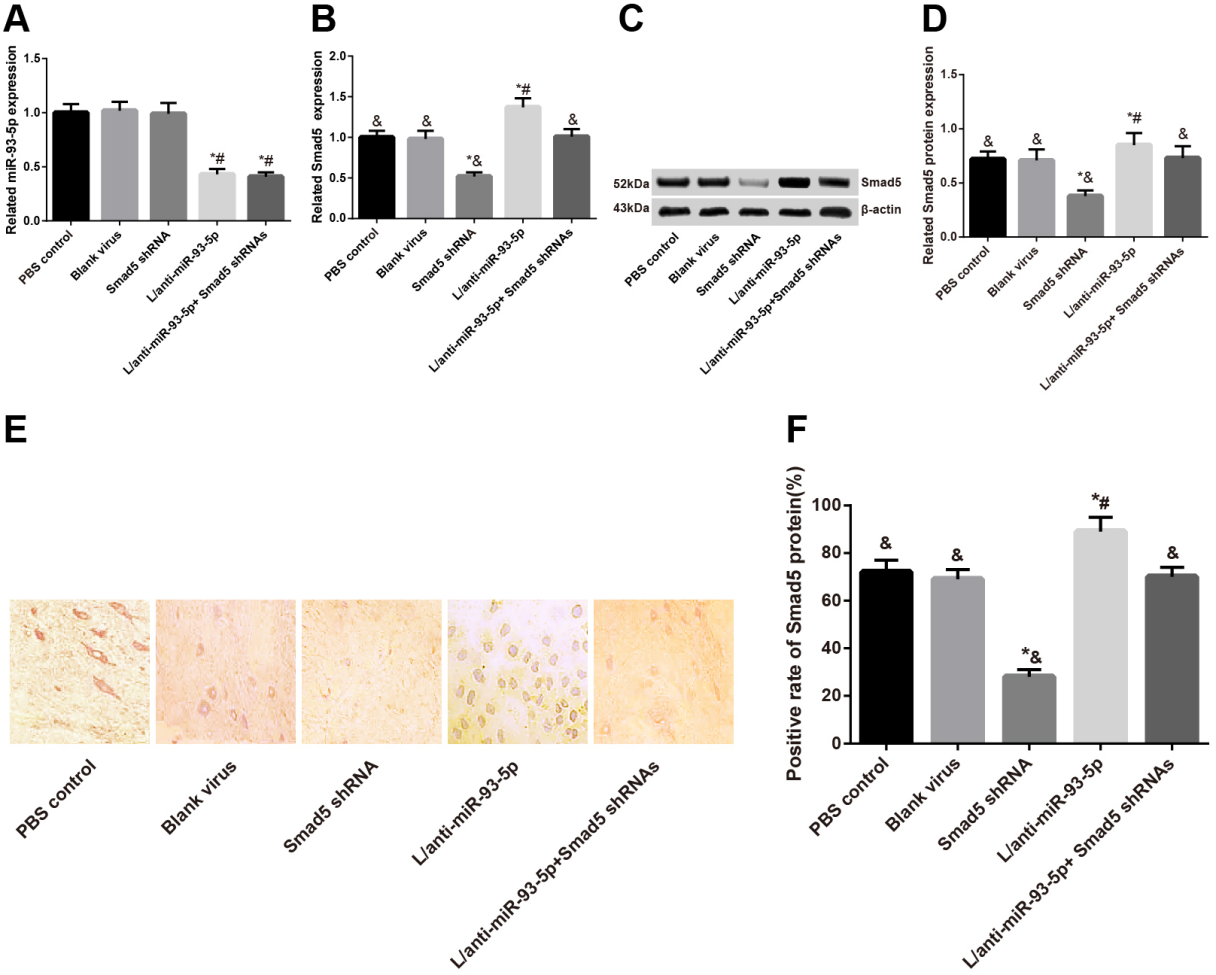
At 14 days after lentivirus transfection, L5 spinal marrows from all five groups were extracted and miR-93-5p expression and Smad5 mRNA and protein expressions were detected **A.** miR-93-5p expression; **B.** Smad5 mRNA expression; **C.** electrophoretogram of Smad5 protein by Western blot; **D.** alternations of Smad5 protein expression; **E.** Smad5 protein expression by immunohistochemistry; **F.** semi-quantitative Smad5 protein expression by immunohistochemistry. Note: *, *P* < 0.05 in comparison with the blank virus group; #, *P* < 0.05 in comparison with the Smad5 shRNA group; &, *P* < 0.05 in comparison with the L/anti-miR-93-5p group.

As shown in Table 2, the L/anti-miR-93 group had the lowest PMWTs, while the Smad5 shRNA group presented the highest PMWTs among these five groups (all *P* < 0.05). No such difference was identified among the L/anti-miR-93-5p + Smad5 shRNA group, the blank group and the PBS control group (all *P* > 0.05). After the last measurement of PMWT at 14^th^ day after injection, the L5 spinal marrow in the mice in the five groups were selected for the observation of virus transfection. As shown in Figure 3, the L/anti-miR-93-5p group, the Smad5 shRNA group and the L/anti-miR-93-5p + Smad5 shRNA group, three of them had a transfection efficiency of about 80%. Meanwhile, using the slices, we detected also miR-93-5p expression and Smad5 mRNA expression with RT-qPCR (Figure 4A-4B) and protein expressionSmad5 with Western Blot Assay and immunohistochemical staining (Figure 4C-4F). We found that the miR-93-5p expression was significantly lower in the L/anti-miR-93-5p group and the L/anti-miR-93-5p + Smad5 shRNA group than that in the other three groups (all *P* < 0.05); however, it presented no difference among the other three groups (all *P* > 0.05). As revealed, both the Smad5 mRNA and protein expressions had the highest value in the L/anti-miR-93-5p group while the lowest value in the Smad5 shRNA group among the five groups (all *P* < 0.05), and no such difference was observed among the L/anti-miR-93-5p + Smad5 shRNA group, the blank group and the PBS control group (all *P* > 0.05).

**Table 2:** The PMWTs of all groups at different timing after lentivirus transfection

	7d	9d	14d
L/anti-miR-93-5p group	3.23 ± 0.25*	3.35 ± 0.31*	3.42 ± 0.35*
Smad5 shRNA group	6.11 ± 0.54^#^	6.24 ± 0.70^#^	6.30 ± 0.75^#^
L/anti-miR-93-5p + Smad5 shRNA group	4.11 ± 0.43^#^*	4.24 ± 0.46^#^*	4.35 ± 0.50^#^*
Blank virus group	4.09 ± 0.38^#^*	4.18 ± 0.49^#^*	4.31 ± 0.51^#^*
PBS control group	4.13 ± 0.51^#^*	4.22 ± 0.45^#^*	4.37 ± 0.55^#^*

PMWT, paw mechanical withdrawal threshold; PBS, phosphate buffered saline

* *P* < 0.05 in comparison with the L/anti-miR-93-5p group

# *P* < 0.05 in comparison with the Smad5 shRNA group.

## DISCUSSION

Usually, the analgesic effect of morphine is realized by upregulation of pain threshold, and patients treated with morphine could endure more intense pain [25]. However, the painkiller effect is decreased along with lasting administration of morphine at a constant dose [26, 27]. In consideration of the proved biological function in bone metastases of mir-93 and Smad5, we hypothesized that miR-93/Smad4 may promote the progression of morphine tolerance in patients with BCP. Our result showed that miR-93 was upregulated while Smad5 was downregulated in the mice with BCP, suggesting that miR-93 was negatively correlated with Smad5. As previous studies suggested, up-regulation of miR-93 was detected in cervical carcinoma by Wang et al., in lung cancer by Du et al., and in head and neck squamous cell carcinoma by Li et al., indicating that miR-93 may contribute to the metastasis and invasion of cancers [28–30]. Also, Kang et al. reported that breast cancer bone metastasis was promoted through the Smad tumor suppressor pathway [31]. Along with statistical analysis, our dual luciferase reporter gene assay, verified that miR-93 could directly suppress the expression of Smad5. With these findings, we go further to investigate the role of miR-93 and Smad5 in the formation of morphine tolerance in BCP mouse model.

As suggested, miR-93 and Smad5 closely correlated with cancer metastasis [30, 32]. When Lewis lung cancer cell metastasize to bone, such algogenic substances as endothelins, prostaglandins, proteases, protons, bradykinin, and tyrosine kinase activators will be released by the cancer cells as well as their associated stromal cells, leading to induction of sensitization and activation of sensory nerves that innervate the bone [33, 34]. In this way, we believed that miR-93 suppressing Smad5 may contribute to the exacerbation of BCP. Using Western Blot assay, RT-qPCR and immunohistochemistry, we found that, in comparison with the healthy mice, the mice with BCP presented differential expression of miR-93 and Smad5. In the bone metastasis, bone damage is not directly caused by cancer/stromal cells but by the receptor activator of nuclear factor κ-B ligand (RANKL) activated by them. The activation of RANKL enhances the binding activity between RANKL and RANK (its receptor), leading to expression of osteoclasts [4, 35]. Aditi Gupta et al. reported that, the expression of RANKL could be knocked down by phosphorylation of Smad5 suppression, therefore reducing osteoclast differentiation and consequently lessening bone loss and BCP [36]. We also learned that activation of PI3Kγ/Akt pathway, downstream of miR-93, could activate spinal microglia through MCP-1 and mediates BCP [37, 38]. Taken together, miR-93/Smad5 is suggested as an important mediator in BCP.

As observed, the mice in the morphine model had a significantly higher miR-93 expression and a lower Smad5 expression than the other groups. After we lentivirus transfection with L/anti-miR-93-5p and/or Smad5 shRNA, we found that the PMWT changed with the expression of miR-93 and Smad5. Our results suggested that downregulation of miR-93 expression in the L/anti-miR-93-5p led to lower PMWTs while downregulation of Smad5 to elevated PMWTs. From this result, we think that miR-93/Smad5 interaction may participate in the formation of morphine tolerance. One possible explanation is that, as BCP progressed, the constant dose morphine fails to produce the analgesic effect as it did at the very start. In addition, Morphine realize it analgesic function trough targeting μ opioid receptor (MOR). Desensitization and trafficking of μ opioid receptor has been widely accepted as a main reason for morphine tolerance [39–41]. By regulating such transcription factors of *OPRM* (MOR gene) as NF-κB through different pathways, miR-93 and Smad5 may participate in the formation of morphine tolerance [36, 42]. In the present study, we only identify a relation of miR-93 and Smad5 to morphine tolerance while leave the mechanism how miR-93 and Smad5 influence morphine tolerance to future study. Above all, our study is a preliminary one. However, these results, if verified with more detailed studies, may be of great significance in the treatment of morphine tolerance. To sum up, we provide direct evidence that Smad5 is a target of miR-93. By regulation Smad5 through miR-93, morphine tolerance may be controlled through regulating BCP or other causative factors.

## MATERIALS AND METHODS

### Ethic statement

This study was conducted following the recommendations proposed by International Association for the Study of Pain. The protocol was approved by the Animal Ethics Committee in Xiangya Hospital and Ningbo No.2 Hospital.

### Construction of L/anti-miR-93-5p lentivirus and Smad5 lentiviral shRNA vector

The present study used the three-plasmid system; three packaging plasmids were pGLV-H1-GFP+Puro, PG-P1-VSVO, PG-P2-REV [20]. The recombinant lentiviral vectors of anti-miR-93-5p and Smad5 shRNA was synthesized by Shanghai GenePharma Co., Ltd and verified through sequencing. The sequence of anti-miR-93-5p was 5′-GAUGGACGUGCUUGUCGUGAAAC-3′ and that of Smad5 shRNA was 5′-AATTACTTTTATTTTGCATTTT-3′. In a 96-well plate, HEK 293T cells were seeded (0.5 × 10^4^ cells/well) and cultured with 10%-fetal bovine serum (FBS) containing DMEM medium (100 μL/well; overnight). On the next day, DMEM containing 10% FBS and polybrene (5 μg/ml) was used to dilute the lentiviruses to concentrations in gradient. After that, the culture solution was removed and replaced with 100 μL lentivirus diluent. Three duplicated wells were set for wells at different dilution ratios of a lentivirus. A blank virus group and a phosphate buffered saline (PBS) control group were built. After overnight incubation, cells continued to be cultured with 100 μL complete medium for 48 hours. Under a micro scope, the GFP-positive cells were counted in each transduction unit (TU) and the titer was calculated as: TU/ml = [(infected cells/field) × (fields/well)]/Volume virus (ml) × dilution factor.

### Animal modeling

From the Nanjing Junke biological engineering co., LTD (Nanjing, China), male C57BL/6 mice (n = 90; age range of 6~8 weeks old; weight range of 18~23 g) were bought. Of 90 mice, 70 were randomly selected for building a BCP model by injecting Lewis lung cancer cells (2 × 10^6^; Shanghai FengShou biological technology co., LTD) suspended with 10 μL PBS into the bone cavity of each mouse [21]. The 50% paw mechanical withdrawal threshold (PMWT) was recorded to decide on the validity of the model before modeling and at 3 days, 6 days and 9 days after modeling [22].

### Animal grouping and preparation of morphine tolerance model

The mice with BCP (n = 70) were randomly allocated into saline model group (n = 10) and morphine model group (n = 60). The 20 healthy controls were contrastively divided into saline control group (n = 10) and morphine control group (n = 10). After 6 days of successfully mouse modeling of BCP, the morphine control group and the morphine model group received hypodermic injection of morphine (10 mg/kg; twice a day) [23]. While the saline model group and the morphine model group had hypodermic injection of normal saline (NS) (10 mg/kg; twice a day). After 6 days of BCP mouse modeling, PMWT was determined at the 1^st^, 3^rd^, 5^th^ and 7^th^ after injection of morphine to confirm whether a morphine tolerance model was successfully built. In each group, 10 mice were killed at 7-day long injection for the detection of miR-93 and Smad5. The other 50 mice of the morphine model group continued to be kept alive until the next experiment.

### Transfection with lentivirus

According to the transfection scheme, the 50 mice of the morphine model group were randomly assigned to L/anti-miR-93 group, Smad5 shRNA group, L/anti-miR-93 + Smad5 shRNA group, blank group (lentivirus-free) and PBS control group. All the five groups were treated with venous injection of corresponding virus suspension or equal sterile PBS solution; all the virus suspensions were of same titer and same dosage. Following the construction of morphine model (at the 7^th^ after injection of morphine), PMWTs at 7, 9 and 14 days after injection were recorded.

### Measurement of PMWT

The measurement of PMWT was conducted based on the method proposed in a previous study [24]. The mice to be tested were placed in a transparent perspex cage (26 cm × 14 cm × 26 cm) and the cage was put on a 22-cm high net rack (each grid of 0.5 cm × 0.5 cm). About 15~20 minutes later, vonFrey cilium (Stoeling, America) of different stresses (0.18, 0.25, 0.6, 1.3, 3.8, 5.4, 7.6 and 9.7 g) was used to poke the mice right at the sensitive postmedian of their paws. A cycle of stimulation at a force consisted of 5 pokes (6~8 seconds/poke). Each poke was conducted at ≥ 2 minutes after the last poke when the mice regain its calmness. The least stress causing ≥ 2 paw withdrawals was described as PMWT.

### Luciferase reporter gene assay

Using PicTar, TagetScan, miRanda and miRDB, the binding site between microR-93-5p and *Smad5* was predicted, finding that 776-782 nt (GCACTTT) at Smad5 mRNA3′-UTR region was the one in search. Based on this finding, we synthesized a target sequence WT (GCACTTT) and then a mutant sequence Mut (CGTGAAA) through site-directed mutagenesis in WT. After receiving digestion with Xho I and Not I restriction enzyme, pmiR-RB-REPORTTM plasmids combined with synthesized WT or Mut to construct recombinant plasmids, pSmad5-WT or pSmad5-Mut. The recombinant plasmids were validated through sequencing. HEK 293T cells were used for cell transfection and luciferase assay. Receiving different transfection schemes, cells were allocated into four groups: Group A, transfected with NC#22 (miR-93-5p negative control) and pSmad5-WT; Group B, transfected with miR-93-5p mimics and pSmad5-WT; Group C, transfected with NC#22 and pSmad5-Mut; Group D, transfected with miR-93-5p mimics and pSmad5-Mut. After transfection, cells were seeded into culture medium-containing (100 μL/well) 96-well plates at a cell concentration of 1.5 × 10^4^ cells/well, followed by incubation in an incubator of saturated humidity (37°C, 5% CO_2_, 24 hours). On the next day, the culture medium was renewed (50 μL/well). Meanwhile, 10 μL reduced serum medium OPTI-MEM (Thermo Fisher Scientific, America) diluting miR-93-5p or NC#22 (100 nmol/L), 15 μL OPTI-MEM diluting pSmad5-WT or pSmad5-Mut (100 ng) and 25μL OPTI-MEM diluting Lipofectamine 2000 (0.25 μL) were intensively mixed together after 5-minute reaction. After 20-minute long incubation at room temperature, the mixture (50 μL) was put into a well for cell incubation. Together with the 50 μL medium, each well contained culture solution of 100 μL. For each group of cells, 3 duplicated well were set. At 6 hours after transfection, the culture solution was renewed again with 100 μL culture medium. At 48 hours after transfection, dual luciferase reporter gene assays were conducted with luciferase reporter gene assay kit (Beyotime Biotechnology, China) and comparisons were made among the four groups in their luciferase activity (hRluc/hLuc).

### Specimen preparation and immumohistochemical staining

After receiving deep anesthesia with Nembutal, mice in the four groups were treated with thoracotomy; a cannula was intubated to the ascending aorta, followed by swill with 0.9% saline (20 mL) to remove the blood in a short time and then perfusion (20 minutes) for fixation with 4% paraformaldehyde-containing phosphate buffer (0.1 mol/L, pH = 7.4). Following the perfusion, L5 spinal marrow was extracted and then fixed (4°C, 4~6 hours). The fixed L5 spinal marrow then was immersed first in 20% and then 30% sucrose solution till it sank to the bottom. After that, the spinal marrow was frozen and then made into serial sections (40-μm). The immumohistochemical staining was conducted with avidin biotin complex (ABC) system. The sections were blocked with 5% goat serum and incubated in rabbit-anti-Smad5 (1:800; ab92698; Abcam, America) first at 37°C for 2 hours and then cultured at 4°C for 48 hours. On the next day, the sections were rewarmed, followed by incubation with Biotin labeled goat-anti-rabbit IgG (37°C, 2 hours) and then with ABC complex (37°C, 1 hours). During the immumohistochemical staining, all the antibodies were diluted with 0.01 mol/L PBS (pH7.4) and the sections were rinsed thoroughly with PBS before they moving on to the next step. After staining, the sections received DAB (short for diaminobenzidine coloration, mounting, drying, dehydration, hyalinization and blockage by gelatin. For the sections of the negative control group, the primary antibody was instituted with PBS. Under OLYMPUS IX81 light microscope (Olympus, Tokyo, Japan; × 200), 10 fields were randomly selected to observe the sections; cells in dark brown were considered as positive. The percentage of positive cells was recorded on a scale of 0 to 3, with 0 indicating less than 10%, 1 representing 10% to 25%, 2 representing 25% to 50%, 3 representing more than 50%. Then, we defined the above results as 0 score (negative), 1~2 scores (weak positive), 3~4 scores (positive) and 5~ scores (strong positive). Finally, 0 score was defined as negative expression and 1~5 scores were defined as positive expression. The positive intensity was analyzed with Image-Pro Plus 6.0 software (Media Cybernetics, Silver Spring, MD, USA).

### Real-time fluorescence quantitative polymerase chain reaction (RT-qPCR)

Through the same method described above, L5 spinal marrow was collected with Diethy pyrocarbonate (DEPC)-containing centrifugal tubes (2 mL), followed by RNA extraction with Trizol (TAKARA, Japan) and measurement of RNA concentration and RNA purification with an μLtraviolet spectrophotometer (Eppendorf, German). A RNA sample of 1000 ng was used for reverse transcription (RT) according to the instruction of PrimeScriptTM RT Regeant Kit (TAKARA, Japan). The reverse transcription system included 5 × PrimeScriptTM Buffer (4 μL), PrimeScriptTM RT Enzyme Mix I (1 μL), 50μM OligodT Primer (1 μL), 100 μM Random 6 mers (1 μL) and RNase Free dH2O (13 μL). The RT protocol was one cycle at 37°C for 30 minutes and then one cycle at 85°C for 6 seconds. The RT-qPCR was conducted with SYBR Premix Ex Taq II kit (TAKARA, Japan). The RT-qPCR system included RT product (1 μL), SYBR Green I Premix Ex Taq II (2 ×) (10 μL), 10 uM PCR Forward Primer (0.8 μL), 10 uM PCR Reverse Primer (0.8 μL), ROX Reference Dye (50×) (0.4 μL) and dH2O (7 μL). The RT-qPCR protocol was one cycle of 30 seconds at 95°C, and then 40 cycles of 5 seconds at 95°C, 30 seconds at 56°C, and 30 seconds at 72°C. The calculation of relative expression of target gene was conducted with 2^−ΔΔCT^ method; the internal reference for miR-93-5p was snRNA U6 and the internal reference for Smad5 was GAPDH. The primer sequences were listed in Table 3.

**Table 3:** List of primer sequences for RT-qPCR

Gene	Forward primer	Reverse primer
MiR-93-5p	5′-AGTCTCTGGCTGACTACATCACAG-3′	5′-CTACTCACAAAACAGGAGTGGAATC-3′
U6	5′-GCTTCGGCAGCACATATACTAAAAT-3′	5′-CGCTTCACGAATTTGCGTGTCAT-3′
Smad5	5′-CTTGGATGGACGTCTGCAAG-3′	5′-CATGGTGAAAGTTGCAGTTC-3′
GAPDH	5′-GCTTGAAGGTGTTGCCCTCAG-3′	5′-AGAAGCCAGCGTTCACCAGAC-3′

RT-qPCR, real-time fluorescence quantitative polymerase chain reaction.

### Western blot assay

The L5 spinal marrow was collected with a DEPC centrifugal tube as described above. After that, 100 μL RIPA lysis buffer (ShineGene Molecular Biotechnology, China) was added to decompose the marrow, followed by homogenate by intensive vibration on ice. At 30 minutes after homogenate, protein extraction was conducted in strict accordance with the instructions. Subsequently, protein measurement was conducted with bicinchoninic acid (BCA) protein quantization kit (Beyotime Biotechnology, China). On basis of the protein concentration, the volume of the upper sample was calculated and the upper sample was mixed with loading buffer at a ratio of 1:1, followed by 5-minute long hot bath and then sample adding. Thus, a sodium dodecyl sulfate- polyacrylamide gel electrophoresis (SDS-PAGE) weldwood was prepared for electrophoresis. The electrophoresis was performed with 5% spacer gel and 12% separation gel first at 80 V and then 100 V. Subsequently, the gel were incised, chamfered and sandwiched, followed by electric transfection (4°C, 200 mA, 2 hours) to a membrane. Next, the membrane received blockage with 5% skim milk (room temperature, 1 hour) and tert-butyldimethylsilyl-Tween (TBST) rinse (10 minutes × 3 times), followed by incubation (4°C, overnight) with monoclonal rabbit-anti-Smad5 antibody (1:1000; ab92698, Abcam, America) or polyclonal rabbit-anti-β-actin antibody (1:4000; ab129348, Abcam, America). On the next day, the membrane was again rinsed (10 minutes × 3 times) and continued to be incubated with HRP-labeled goat-anti-rabbit IgG at room temperature for 1 hour (Sigma, America). After that, the membrane was rinsed for coloration with an ECL kit and scanned with a gel imaging system. For analysis on the target bands, ImageJ software was used. In the calculation of Smad5 expression, β-actin was the internal reference.

### Statistical analyses

For data analyses, SPSS21.0 was used (SPSS Inc., Chicago, IL, USA). Measurement date was expressed as mean ± standard deviation. Among-group comparisons were conducted by variance analysis and between-group comparisons were by t test. Pearson analysis was used for correlation analysis. All the tests were two-tailed; *P* < 0.05 indicated significant difference.
